# Screening of Cognitive Function and Hearing Impairment in Older Adults: A Preliminary Study

**DOI:** 10.1155/2014/867852

**Published:** 2014-07-22

**Authors:** Lena Lar Nar Wong, Joannie Ka Yin Yu, Shaina Shing Chan, Michael Chi Fai Tong

**Affiliations:** ^1^Division of Speech & Hearing Sciences, Faculty of Education, The University of Hong Kong, Pokfulam Road, Hong Kong; ^2^Department of Otorhinolaryngology, Head and Neck Surgery, The Institute of Human Communicative Research, The Chinese University of Hong Kong, Hong Kong

## Abstract

*Background*. Previous research has found that hearing loss is associated with poorer cognitive function. The question is that when a hearing impairment is being compensated for by appropriately fitted monaural hearing aids, special precautions are still needed when screening cognitive function in older adults. *Objective*. This research examined cognitive function in elderly hearing aid users who used monaural hearing aids and whether the presence of a hearing impairment should be accounted for when screening cognitive function in these individuals. *Methods*. Auditory thresholds, sentence reception thresholds, and self-reported outcomes with hearing aids were measured in 34 older hearing aid users to ensure hearing aids were appropriately fitted. Mini-Mental State Examination (MMSE) results obtained in these participants were then compared to normative data obtained in a general older population exhibiting similar demographic characteristics. Stepwise multiple regression analyses were used to examine the effects of demographic and auditory variables on MMSE scores. *Conclusions*. Results showed that, even with appropriately fitted hearing aids, cognitive decline was significant. Besides the factors commonly measured in the literature, we believed that auditory deprivation was not being fully compensated for by hearing aids. Most importantly, screening of cognitive function should take into account the effects of hearing impairment, even when hearing devices have been appropriately fitted.

## 1. Hearing Loss and Cognitive Function

Besides hearing impairment, decline in cognitive functions is also commonly observed in the aging population. Recent studies showed that reduced auditory input due to a hearing impairment is also associated with greater declines in cognitive function in older adults than those without hearing loss.

Lin and colleagues [1] showed that older adults with hearing impairment would have a 24% increased risk for declines in cognitive function over time and may experience a 30 to 40% faster decline than those without a hearing loss. Furthermore, this decline was related to the degree of hearing loss measured at baseline. Lin [2] further reported that a mild to moderate hearing impairment in adults aged 60 to 69 years was associated with poorer executive function and psychomotor processing, while Lin et al. [3] reported that greater hearing loss in older adults was associated with not only lower scores in memory test, but also poorer mental status and executive function, such as shifting attention and inhabiting. Similar findings were reported by Wingfield and Tun [4] that those with a mild to moderate hearing loss had greater difficulties with recall, which could be a reflection that effortful listening took away resources available for storing information in working memory. Tay et al. [5] also found that, among adults of 50 years of age and over, those with a moderate to severe hearing loss exhibited slightly poorer Mini-Mental State Examination (MMSE) score than those with normal hearing. Results from Lindenberger and Baltes [6] and Schneider et al. [7] concur with these findings.

Various hypotheses have been proposed to explain this decline associated with hearing impairment. While there could be a common neuropathologic origin that underlies both hearing and cognitive decline, the hearing loss could possibly lead to a cycle of multimorbidity in different areas or may interact with other risk factors to accelerate cognitive declines [1]. The hearing impairment may also result in a deprivation of auditory inputs, leading to structural or functional changes related to cognitive function [8, 9]. Trying to fill in the gaps caused by missing speech information may result in a shortage of resources for information encoding and storage in an already reduced working memory in older adults [7, 9–11]. With greater hearing loss, speech understanding is more likely to be adversely affected. Speech understanding becomes effortful, resulting in withdrawal from social interactions, which could precipitate further cognitive declines [8]. Finally, some studies have shown that tests that are administered auditorily may show a cognitive deficit because older individuals are being disadvantaged by their disability [9]. One important question, therefore, is whether older adults with appropriately fitted hearing aids are able to demonstrate cognitive ability comparable to that of the general population when cognitive measures are being administered using verbal instructions.

## 2. Hearing Aid and Cognition

A search of the literature revealed only a small number of studies that had examined the effects of hearing aid use on cognitive function in the elderly population; findings were however inconclusive. For example, in a randomized control trial, Mulrow et al. [13] found that cognitive function measured on the Short Portable Mental Status Questionnaire (SPMSQ) improved after four months of hearing aid use in a group of older adults (mean age above 70 years, *n* = 13) with an average hearing loss of about 50 dB HL. Using the MMSE, Acar et al. [14] also showed significant improvement in cognitive function in a group of elderly subjects (mean age about 70 years) after three months of hearing aid use. However, because the cognitive function tests in both studies were administrated verbally prior to hearing aid fitting, the reduced cognitive function could have been confounded by the hearing disability.

While Lin [2] also found that the use of hearing aids was positively associated with cognitive function, Young Choi et al. [15] demonstrated significant changes in the total scores measured on the visual verbal learning test (VVLT) after six months of hearing aid use, compared to a control group of nonusers. Due to the small sample sizes, the findings in these studies should be interpreted with some caution.

On the contrary, other studies were not able to demonstrate improved cognitive function after six to 12 months of hearing aid use. Tesch-Römer [16] was not able to find changes in executive function and memory after six months of hearing aid use by those with a mild to moderate hearing loss. They attributed the lack of changes to subjects not being randomly assigned and six months of hearing aid use being too short to cause a significant change. In another study, Van Hooren et al. [17] evaluated cognitive function in terms of processing speed, reasoning, memory, knowledge, and verbal fluency after 12 months of HA use. No improvement was observed, compared to a control group of non-hearing aid users.

In a literature review of relevant studies, Kalluri and Humes [18] pointed out that there was a lack of strong evidence on the long-term effects of hearing aids on cognition. Furthermore, given that many older adults did not pursue intervention for 8 to 12 years after the first notice of a hearing impairment [17], a longer duration of hearing aid use is probably needed to demonstrate any effects of reversal of cognitive decline [16]. Among the majority of the studies, there was also a lack of information on whether amplification was well fitted and therefore it was uncertain whether the deficit in hearing had been appropriately compensated for.

Given the limitations associated with previous research, the present study controlled for the effects of hearing aids by documenting whether they have been appropriately fitted and administering the cognitive function tests with hearing aids at optimal settings. The subject sample was typical of the vast majority of hearing aid users in Hong Kong and in many developing countries, where, due to low income, they have opted for monaural hearing aids. They also exhibited poorer hearing than hearing device users in Western societies because Hong Kong Cantonese speakers were often not motivated to seek help until their hearing loss has exceeded 40 dB HL [18]. More severe hearing loss is related to distortion in hearing that could not be fully compensated for by the use of hearing aids and may precipitate greater declines in cognitive function [3]. The severity of hearing loss is such that the unaided side was also being deprived of auditory inputs. Thus, the characteristics of our subject sample were such that we expected to observe a decline in cognitive function. Our results on the cognitive function test were compared with norms obtained on the general older population with similar demographic characteristics [12]. Whether cognitive function was related to demographic (i.e., age and gender) and auditory variables (i.e., pure tone thresholds and speech reception thresholds) was examined. These results may have important implications on the screening and diagnosis of cognitive decline in those with a hearing impairment.

## 3. Methodology

### 3.1. Participants

A total of 34 hearing impaired elderly Cantonese speakers, aged above 60 years and exhibiting a bilateral mild to severe degree of hearing loss, regardless of the nature of the loss, were recruited. They were current users who had been wearing a monaural hearing aid for at least one year, to allow for adaption to amplification. Consecutive medical records at the Audiological Centers at the Prince of Wales Hospital (PWH) and Alice Ho Miu Ling Nethersole Hospital were reviewed for subject recruitment. Elderly individuals with reportedly poor physical or mental health, non-Cantonese speakers, and those not meeting the inclusion criteria were excluded.

Among the participants, 73.5% were married, 5.9% were single, 17.6% were widowed, and 2.9% were divorced/separated. In terms of other otologic conditions, 52.9% reported experiences of tinnitus, 21.2% had dizziness, and 32.4% had noise exposure. In terms of health issues, 8.8% reported having diabetes, 52.9% had high blood pressure, and 17.6% had heart problems but, overall, 88.2% of the participants reported average to good health; only 11.8% reported poor health. Participants were all community dwellers.

**Figure 1:**
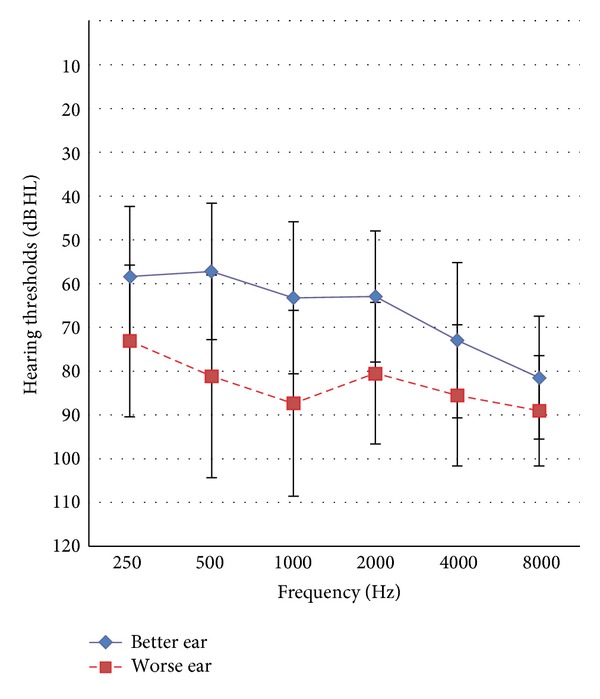
Audiometric thresholds are shown with standard deviations as error bars.

Table 1 shows other demographic information of the participants, suggesting that on average they had a lower secondary education. Without hearing aids, these subjects exhibited a hearing loss of 64.9 dB HL (SD = 15.2), averaged at 500, 1000, and 2000 Hz in the better ear and 80.7 dB HL (SD = 16.2) in the worse ear (see Figure 1). While on average participants had noted a hearing impairment for almost 17.8 years, they had only worn hearing devices for the past 6.9 years. In other words, they had waited for about 11 years before obtaining intervention. Table 1 also shows demographic characteristics of the normative sample for comparison with results obtained in the present study.

### 3.2. Materials and Equipment

All of the audiometric testing was conducted using a GSI 61 audiometer. Ear specific unaided air conduction thresholds at 0.25, 0.5, 1, 2, 4, and 8 kHz and ear specific bone conduction thresholds at 0.5, 1, 2, and 4 kHz were obtained in both ears using a TDH-49 headphone. Soundfield audiometric thresholds were obtained using warble tones at 250, 500, 1 k, 2 k, 3 k, and 4 kHz in both the aided and unaided conditions.

The Cantonese Hearing In Noise Test was used to measure speech recognition in quiet and in three noise conditions [19]. The noise conditions were (1) speech front (SF), where speech and noise were both presented from the front speakers; (2) noise on the hearing aid side (N-HA), where speech was from the front and noise was from the side where the hearing aid was worn; and (3) noise on the non-hearing aid side (N-NA), where speech was from the front but noise was being presented on the side where a hearing aid was not worn. The speech was adjusted adaptively, based on the correctness of the responses. For testing in noise, the noise level was fixed at 65 dB(A). The speakers were placed at one meter away from the center of the head of the subjects. The HINT system was used to present the stimuli and score the responses. A sentence recognition threshold (SRT) in quiet was defined as the signal level, in dB(A), where a participant is able to repeat 50% of the sentences correctly. A SRT in noise was defined as the signal-to-noise ratio (in dB* S/N*), where the participant is able to repeat 50% of the sentences correctly. Aided and unaided SRTs were obtained. Audiological assessment and the CHINT were conducted in a sound treated room at the Audiology Centre of the PWH that met the ANSI S3.1-1991 standard for maximum permissible ambient noise levels.

Participants also filled in the Chinese version of the International Outcome Inventory of Hearing Aid (IOI-HA) to evaluate self-reported outcomes with hearing aids in seven domains: usage, benefit, residual activity limitation, satisfaction, residual participation restriction, effects of the hearing impairment on significant others, and quality of life [20]. A maximum score of 5 is possible for each domain, which indicates the best outcomes possible with amplification.

The audiological assessment, SRTs, and IOI-HA were administered to ensure that the hearing aids were fitted properly and provided optimal outcomes. The MMSE was conducted to assess cognitive function [21]. The MMSE evaluates cognitive function in the following domains.

1. “Orientation to time” measures the participant's sense of date and time, to yield a maximum score of five points.
2. “Orientation to place” evaluates the patient's sense of location, to yield a maximum score of five points.
3. “Registration” accesses the ability to repeat a short list of common items and a maximum score of three points is possible.
4. “Attention and calculations” evaluates arithmetic ability by having the patient count backward from 100 using a step size of 7; a maximum score of five is possible.
5. “Recall” evaluates whether the individual is able to recall the items from “Registration,” to yield a maximum score of three.
6. “Language” measures whether the patient could name two common objects; a maximum score of two points is possible.
7. “Repetition” involves having the individual repeat a short phrase; one point is given for a correct response.
8. “Complex Commands” involves having the individual follow instructions to perform a task or draw, to yield a maximum score of six points.

The total MMSE score ranges from 0 to 30 and the MMSE took less than 10 minutes to be completed. The Cantonese version of the MMSE [22] was administered with the hearing aids of the participants adjusted to a level optimal for speech understanding in a quiet sound treated room.

### 3.3. Procedures

Research ethics were approved by the Joint Chinese University of Hong Kong—New Territories East Cluster Clinical Research Ethics Committee (CREC) (Ref. number CRE-2013.481). Informed consent was obtained at the start of the data collection process. Demographic data, including age, gender, marital status, educational level, hearing and otological history, and medical history, were documented. Electroacoustic measurements were conducted on the hearing aids to ensure they were in proper working order. Hearing aids were adjusted once to settings optimal for speech understanding, prior to administering tests. Hearing assessments, cognitive assessments, and the IOI-HA were then administered in random orders. The testers took special care (e.g., repetition, speaking at a slower rate, and enunciating each word clearly) to ensure that the test instructions were heard clearly. The whole test procedure took approximately two hours. To avoid fatigue, breaks were given to subjects after one hour of assessment or upon their request. A transportation allowance of HKD 200 (or USD 25) was provided.

## 4. Results

Soundfield pure tone average hearing thresholds improved significantly from 62.7 dB HL (SD = 13.6) unaided to 41.8 dB HL (SD = 7.3) aided; *t*(31) = 11.3; *P* < .001; Cohen's *d* = 2.4. With hearing aids, a significant improvement, with small to large effect sizes, in SRTs obtained in quiet and in all three noise conditions was noted (see Table 2). The improvement in SRT was the greatest when the noise was presented to the side of the nonaided ear and speech was from the front (N-HA condition).

**Figure 2:**
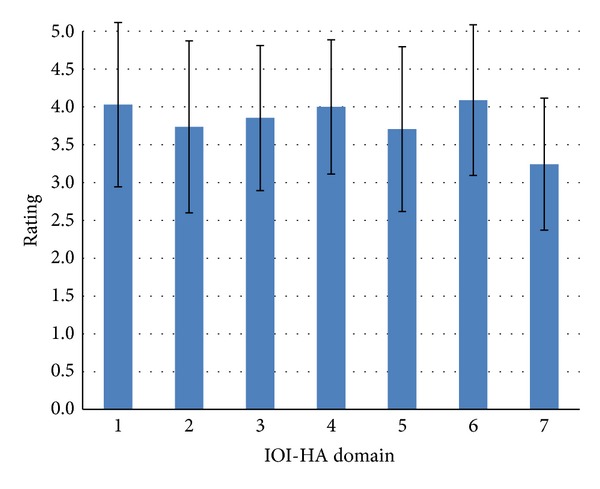
Mean IOI-HA ratings are shown with standard deviations as error bars. The respective domains are 1 = usage, 2 = benefit, 3 = residual activity limitation, 4 = satisfaction, 5 = residual participation restriction, 6 = effects of the hearing impairment on significant others, and 7 = quality of life. A maximum rating of 5 indicates best outcomes with the respective domain.

Results from the IOI-HA are reported in Figure 2 and suggest that these subjects used hearing aids for an average of about 4 to 8 hours per day. The ratings for other items ranged from 3.71 to 4.09, out of a maximum of 5, suggesting that these participants were on average obtaining quite a lot of benefit, were experiencing slight difficulty with hearing, and thought that hearing aids were worth the trouble, that hearing difficulties had affected their life slightly, and that significant others were only slightly bothered by the hearing impairment. The ratings on quality of life were lower (mean rating of 3.24, out of a maximum of 5), suggesting that hearing aids have made their enjoyment of life slightly to quite a lot better.

Table 1 shows that the demographic characteristics of the subjects in the current study are quite similar to those obtained on the general older population in Hong Kong, providing justification for their results to be compared.  Table 3 shows the results from the MMSE. An independent samples *t*-test revealed a significant difference in MMSE total scores; *t*(72) = −3.18; *P* < .005; Cohen's *d* = .72. Domain scores could not be compared because of a lack of normative data.

As MMSE total scores were lower among those with hearing impairment and using hearing aids, we attempted to explore whether cognition was related to demographic and auditory variables. These variables included age, gender, aided soundfield average hearing thresholds, duration of hearing loss, duration of hearing aid use, and aided SRTs obtained in quiet and in noise as a composite score. The noise composite SRT was calculated using the following formula: [(noise front SRT × 2) + noise hearing aid side SRT + noise non-hearing aid side SRT]/4 [19]. Stepwise multiple regression analyses were conducted with these as auditory variables and MMSE total and domain scores as dependent variables.  Table 4 shows the results and indicates that only five of the eight MMSE domains were predicted by one of the auditory variables, with small to medium effects sizes. That is, Orientation to time and place could be predicted by noise composite SRT; aided soundfield thresholds predicted scores on Registration and Complex Commands. Duration of hearing aid use contributed to scores on Repetition. Other regression models were not significant.

## 5. Discussion

Overall, results from audiological assessment and the CHINT suggest that hearing aids brought significant benefits in terms of improving sensitivity to sounds and speech reception. Results from the IOI-HA also revealed that participants were using their hearing aids consistently and reported very positive outcomes with the hearing aids. In fact, patient records showed that their hearing aids were adjusted to the satisfaction of the users and further adjustment was not needed. Thus, we were ensured by these results that the hearing aids had been appropriately fitted.

Results obtained on the MMSE showed slight but significant decline in overall cognitive function (with moderate to large effect size) among the subject population. Interestingly, out of the eight domains measured on the MMSE, auditory factors (duration of hearing aid use, aided noise composite SRTs, and aided soundfield thresholds) predicted the scores on five MMSE domains that required understanding of the verbal instructions. Auditory or demographic variables did not predict scores measured on Attention and calculations, Recall, or Language. Nonetheless, these findings suggested that hearing and cognition are intricate aspects of the aging process.

While the current study could not delineate which of the hypotheses mentioned above made greater contribution to the results, we would elaborate on three issues here. First, there was deprivation of auditory inputs in the participants. As mentioned above, the users waited an average of 11 years before they obtained hearing aids. The long-term deprivation in auditory inputs prior to hearing aid fitting might not be fully reversible even with the use of hearing aids. Similarly, monaural hearing aid use might have resulted in the unaided ear being deprived of auditory inputs. Although it would be difficult to control the duration of the wait to get intervention, future research could compare cognitive outcomes between monaural and binaural users.

Second, one could argue that more gain could be provided to further optimize the hearing. Although the participants felt that their hearing aids were fitted appropriately, aided hearing thresholds obtained in the soundfield were improved to 42 dB HL, which meant that some of the weaker signals were not audible. When the hearing disability is not being fully compensated for, the efforts spent on understanding conversations may result in fewer resources being left for storing information [23]. Listening in noise is particularly difficult when extra resources have to be allocated to make up the missing speech information that was being masked. However, increasing gain was not considered appropriate among the participants, as patient records showed that they were not able to tolerate further increase in gain.

Finally, we could not rule out the possibility that some participants might have difficulties understanding the verbal instructions. While there were several measures to ensure optimal understanding of the instructions during cognitive testing, it would be difficult to rule out the possibility that some instructions were not heard clearly. As discussed above, auditory factors seemed to influence scores on the MMSE domains that required understanding of the verbal instructions, although the effect sizes are only small to medium. Schneider and Pichora-Fuller [8] also found that when visually administered cognitive tests were used, hearing impairment did not relate to reduction in cognitive function and therefore should be used as much as possible when screening and diagnosing cognitive decline. Future studies should therefore utilize cognitive measures that minimize the need to listen.

### 5.1. Implications on Clinical Practice

The present and previous researches have shown that hearing aid users tend to wait a long time before they take up hearing devices [17]. As hearing loss is related to cognitive declines [3], it would be crucial for doctors, healthcare workers, and others who work closely with older adults to encourage them to try hearing aids early. We hope that the use of hearing devices could at least slow down, if not arrest, this decline. A longitudinal study is required to examine the progression of cognitive function and provide evidence to the use of hearing devices. In addition, as mentioned above, clinicians should be aware of the implication of a hearing impairment on cognitive function even when appropriate monaural hearing devices are worn.

Emerging evidence is showing that hearing aid users with poorer cognitive function are less able to take advantage of more advanced signal processing algorithms that are supposed to aid speech understanding. These may include noise reduction and compression with short time constants and directional microphones [23–26]. Therefore, Lunner et al. [23] argued that hearing aid fitting should be individualized to release working memory resources, in order to maximize hearing potentials. While a “cognition-driven signal-processing” hearing aid has yet to become a reality, clinicians should not assume that all older individuals would be able to benefit from these algorithms.

In Hong Kong, it is uncommon for individuals with hearing impairment to receive aural rehabilitation, other than the fitting of amplification devices. Via a meta-analysis, Chisolm and Arnold [27] have shown that auditory perceptual training could enhance short-term outcomes with hearing aids. Furthermore, cognitive training has also been shown to improve cognitive function [28] and Kwok et al. [29] were able to show similar training effects, as well as improvement in mental health among community-dwelling Chinese older adults in Hong Kong. Knowing that hearing loss may have concomitant effects on cognition, clinicians and policy makers should consider adding these components to intervention.

We reported findings from a preliminary study and they are somewhat limited by the small sample size. However, the results will help us plan follow-up studies that address the imminent issues. A larger scale study is being carried out in our laboratory to examine the application of several cognitive tests in the subject population. The current study took a cross-sectional view of cognitive function in a general hearing aid user population; a carefully planned longitudinal study would hopefully help establish a causal relationship between the long-term use of hearing aids that are appropriately fitted and cognitive function. Learning about whether hearing aid use could reverse or arrest the progression of cognitive decline is essential for clinicians to make evidenced based recommendation on early hearing aid use. It will also be interesting to find out whether other invention options, such as the use of binaural hearing aids and perceptual and cognitive training, could improve cognitive function to a level commensurate with that of the general older population.

## 6. Conclusion

Overall, the present study showed that while appropriately fitted monaural hearing aids could partially make up for the hearing disability and improve speech understanding, the use of hearing aids may not fully compensate for the decline in cognitive function associated with hearing loss. Therefore, when screening cognitive function, the presence of a hearing impairment should be accounted for. In particular, ensuring audibility of signals and perhaps the use of cognitive function tests that employ visual presentation of stimuli should be used. We have also identified a few research areas where greater understanding on the relationship between cognition and hearing impairment would improve the clinical management of older patients.

## Figures and Tables

**Table 1:** Demographic variables of the subjects in the present study and the comparison reference population in Wong et al. [12].

Demographic variables	Present study	Wong et al. [12]
Age (years)	69.9 (5.6)	69.2 (7.2)

Gender (*n*)	15 male	20 female
	19 female	20 male

Educational level (years)	7.3 (3.5)	7.3 (4.5)

Duration of hearing loss (years)	17.8 (16.5)	N/A

Duration of hearing aid use (years)	6.9 (4.3)	N/A

**Table 2:** Mean sentence reception thresholds (SRTs) and the standard deviations (in brackets). Paired sample *t*-tests were conducted to compare SRTs obtained in the aided and unaided conditions; statistically significant differences between aided and unaided SRTs were found in all test conditions (*P < .001, 2-tailed).

Test conditions	Unaided	Aided	*t*-statistics	Cohen's *d*
Quiet (dB A)	68.9 (9.9)	59.7 (10.3)	6.0∗	1.05

Noise front (NF) (dB *S*/*N*)	8.9 (4.7)	6.0 (4.7)	5.6∗	.77

Noise on the hearing aid side (N-HA) (dB *S*/*N*)	8.3 (6.4)	6.4 (5.2)	2.9∗	.36

Noise on the non-hearing aid side (N-NA) (dB *S*/*N*)	8.2 (5.5)	4.6 (5.7)	4.0∗	.62

**Table 3:** Results on the MMSE from the present study and the comparison reference population in Wong et al. [12].

	Present study	Wong et al. [12]
Orientation to time	4.8 (.50)	N/A
Orientation to place	4.7 (.65)	N/A
Registration	2.8 (.53)	N/A
Attention and calculations	3.7 (1.69)	N/A
Recall	1.6 (1.12)	N/A
Language	1.9 (.17)	N/A
Repetition	.97 (.17)	N/A
Complex Commands	5.5 (.66)	N/A

Total score	26.4 (3.09)	28.2 (1.5)

**Table 4:** Results from stepwise linear regression analysis. The dependent variables (DV) included age, gender, aided soundfield average hearing thresholds, duration of hearing loss, duration of hearing aid use, and aided speech reception thresholds (SRTs) obtained in quiet and in noise as a composite score. Only models with statistical significance are listed with their dependent variables and variables entered into the model (EV).

Models tested	*R* square	*F*-statistics	Beta	*t*-statistics	Cohen's *f* ^2^
DV: Orientation to time	.21	7.2∗	−.47	−2.7∗	.27
EV: noise composite SRT					

DV: Orientation to place	.16	5.2∗	−.41	2.3∗	.19
EV: noise composite SRT					

DV: Registration	.27	9.9∗∗∗	.52	3.1∗∗∗	.37
EV: aided soundfield thresholds					

DV: Complex Commands	.24	8.1∗∗	−.48	−2.8∗∗	.32
EV: aided soundfield thresholds					

DV: Repetition	.33	12.8∗∗∗	−.58	−3.6∗∗∗	.49
EV: duration of hearing aid use					

Note: *P < .05, **P < .01, and***P < .005.
